# Systems metabolic engineering of *Escherichia coli* for the bioproduction of biliverdin and phycoerythrobilin

**DOI:** 10.3389/fpls.2025.1640158

**Published:** 2025-07-25

**Authors:** Shuang Li, Chang Lu, Xiaonan Zang, Delin Duan, Zhanru Shao

**Affiliations:** ^1^ CAS and Shandong Province Key Laboratory of Experimental Marine Biology, Center for Ocean Mega-Science, Institute of Oceanology, Chinese Academy of Sciences, Qingdao, China; ^2^ Laboratory for Marine Biology and Biotechnology, Qingdao Marine Science and Technology Center, Qingdao, China; ^3^ University of Chinese Academy of Sciences, Beijing, China; ^4^ Yantai Key Laboratory of Characteristic Agricultural Bioresource Conservation & Germplasm Innovative Utilization, School of Life Sciences, Yantai University, Yantai, China; ^5^ Key Laboratory of Marine Genetics and Breeding (Ocean University of China), Ministry of Education, Qingdao, Shandong, China

**Keywords:** biliverdin, phycoerythrobilin, heterologous expression, biosynthesis, neoporphyra haitanensis

## Abstract

Phycobiliprotein is an important co-pigment in photosynthesis, which is composed of the covalent combination of apoprotein and phycobilin. Biliverdin IXα and phycoerythrobilin are both important substances in the phycobiliprotein biosynthesis pathway. As an economic red seaweed, *Neoporphyra haitanensis* has a high content of phycoerythrin. Therefore, in this study, we explored new enzyme resources for the heterologous biosynthesis of biliverdin and phycoerythrobilin. Specifically, we identified and isolated the genes encoding NhHO1, NhPebA and NhPebB from *N. haitanensis*, which are integral components of its phycoerythrin biosynthetic pathway. Additionally, *ApHO1* from *Arthrospira platensis* and *PebS* from *Prochlorococcus* phage P-SSM2 were selected for comparative analysis. The results showed that genes from *N. haitanensis* did not encode active enzymes, which might be ascribed to the absence of crucial motifs. However, the transformation of *ApHO1* and *PebS* into *Escherichia coli* could lead to the synthesis of biliverdin and phycoerythrobilin. This is the first report of sequence analysis and enzyme activity verification of phycoerythrin synthesis genes from *N. haitanensis*, providing a foundation for future explorations into its potential genetic resources. The successful production of biliverdin and phycoerythrobilin lay a foundation for the environmentally friendly preparation of phycobiliprotein.

## 1 Introduction

Phycobilisome (PBS) was first discovered by Gantt and Conti (1966). In cyanobacteria and red alga, PBS primarily mediates light harvesting functions associated with Photosystem II (Adir, 2005). PBS consists of phycobiliproteins (PBPs), which are covalently linked to linear open chain tetrapyridine (phycobilins), and linker proteins (Li et al., 2023). Except in photosynthesis, PBPs have been widely used in the fields of food, cosmetics, fluorescent probes, pharmaceuticals and biomedicine (Ji et al., 2023). Currently, PBPs are mainly extracted from *Spirulina* through complex steps (Li et al., 2023). The extraction of PBPs can be devided into physical (Mittal et al., 2017), chemical (Tavanandi et al., 2019) and biological (Mittal and Raghavarao, 2018) methods (Ji et al., 2023). The extracted crude protein needs complex post-processing of further separation, concentration and purification (Ma et al., 2003; de Amarante et al., 2020; Ji et al., 2023). Therefore, the biosynthesis of PBPs and phycobilin in chassis cells (e.g. *E. coli* and cyanobacteria) has attracted much attention (Ge et al., 2018; Li et al., 2023). Aminolevulinic acid (ALA) is converted to protoporphyrin by the multistep reactions, followed by the production of heme (Li et al., 2023). Heme is then subjected to catalytic cleavage by heme oxygenase (HO) to yield biliverdin IXα (BV) (Dammeyer and Frankenberg-Dinkel, 2008). BV is eventually catalyzed by ferredoxin-dependent bilin reductases (FDBRs) to be converted into phycoerythrobilin (PEB) or phycocyanobilin (PCB) (Li et al., 2023).

BV is produced by heme ring cleavage catalyzed by HO (Chen et al., 2012). During this process, HO catalyzes the conversion of heme into BV, along with the generation of carbon monoxide (CO) and ferrous iron (Fe^2+^), (Sugishima et al., 2019). In cyanobacteria, red algae and plants, BV mainly acts as a precursor of phycobilins (Chen et al., 2012). It acts with biliverdin reductase, which can be used as a potential anti-inflammatory therapeutic agent (Florczyk et al., 2008; Soares and Bach, 2009; Chen et al., 2012; Wegiel and Otterbein, 2012). Currently, BV is prepared by oxidizing bilirubin, but the cost is very high (Mei et al., 2024). As a result, there is an urgent need to develop cost-effective and scalable methodologies for BV production. In recent years, synthetic biology technology has received extensive attention, and studies on biosynthesis of BV have been reported. Based on the expression of HO gene in cyanobacteria, Chen et al. (2012) developed a method for large-scale production, recovery and purification of BV from *E. coli*. In addition to *E. coli*, Seok et al. (2019) reported the production of biliverdin using *Corynebacterium glutamicum* as a biological system.

PEB is an open-chain tetrapyrrole derived from heme (Stiefelmaier et al., 2018). There are two ways to generate PEB from BV. The first pathway involves two sequential reduction steps, 15,16-dihydrobiliverdin:ferredoxin oxidoreductase (PebA) catalyzes the reduction of BV to 15, 16-dihydrobiliverdin (DHBV), which is subsequently converted into PEB via the action of phycoerythrobilin:ferredoxin oxidoreductase (PebB) (Dammeyer and Frankenberg-Dinkel, 2006; Aras et al., 2020; Heck et al., 2024). Recent studies have also identified a novel enzyme, phycoerythrobilin synthase (PebS), belonging to the FDBRs family, which catalyzes reactions from BV to PEB independently (Dammeyer et al., 2008; Heck et al., 2024). PEB has high application value, as a natural non-toxic colorant, in food and cosmetics (Stiefelmaier et al., 2018). Currently, pure PEB needs to be obtained by boiling red algal cells in methanol and further liquid chromatography purification steps (Stiefelmaier et al., 2018). Therefore, PEB also needs to develop an efficient production method. Obviously, the research on synthetic biology of PEB is particularly important. Recent studies have demonstrated the feasibility of PEB biosynthesis in *E. coli* through heterologous gene expression (Stiefelmaier et al., 2018).

In this study, the genes (*HO1*, *PebS*, *PebA* and *PebB*) from *Neoporphyra haitanensis*, *Arthrospira platensis*, and *Prochlorococcus* phage P-SSM2 were selected for investigation. The corresponding genes were transferred into *E. coli* to achieve the synthesis of BV and PEB. In this study, the functions of key enzymes in the biosynthesis of phycoerythrin (PE) in *N. haitanensis* were analyzed, which is helpful to further explore the key genes affecting the quality of *N. haitanensis*. At the same time, the selection and verification of genes from diverse organisms can provide a new reference for the biosynthesis of PE.

## 2 Materials and methods

### 2.1 Sample collection

The *N. haitanensis* samples were collected from Putian, Fujian and Dafeng, Jiangsu, respectively. The samples were washed three times with sterile ddH_2_O, and the surface moisture was absorbed with gauze in prior to frozen in liquid nitrogen and stored at -80°C.

### 2.2 Cloning of NhHO1, ApHO1, NhPebA, NhPebB and PebS genes

Total RNA of *N. haitanensis* was extracted with the Plant RNA Kit (OMEGA, USA), and then the RNA was converted into cDNA according to SPARKscript II RT Plus Kit (Sparkjade, China). The candidate NhHO1, NhPebA and NhPebB genes were retrieved from the transcriptome database of *N. haitanensis*. The RACE clone was used to obtain the complete ORF region of these three sequences. The correctness of these sequences was confirmed by comparing with the sequences retrieved from the genome of *N. haitanensis*, which was provided by Professor Wang Dongmei of Ocean University of China (OUC) (**Supplementary Table S1**) (Cao et al., 2020). Heme oxygenase from *A. platensis* (ApHO1) was provided by Professor Zang Xiaonan of OUC. PebS of *Prochlorococcus* phage (GenBank accession: Q58MU6.1) was downloaded from NCBI.

### 2.3 Sequence analysis of NhHO1, ApHO1, NhPebA, NhPebB and PebS genes

The five obtained coding sequences were translated into amino acid sequences with ORF Finder (https://indra.mullins.microbiol.washington.edu/sms2/orf_find.html). The sequences were then aligned by CLUSTALW (https://www.genome.jp/tools-bin/clustalw), and the results was illustrated by ESPript (https://espript.ibcp.fr/ESPript/cgi-bin/ESPript.cgi). The secondary structures were predicted by NPS@ (https://npsa.lyon.inserm.fr/cgi-bin/npsa_automat.pl?page=/NPSA/npsa_sopma.html). The physical and chemical parameters (molecular weight, isoelectric point) of the five proteins were predicted with ProtParam (https://web.expasy.org/protparam/). The SignalP v4.1 Server (https://services.healthtech.dtu.dk/services/SignalP-4.1/) was used to predict signal peptides. The transmembrane helices were predicted with the TMHMM Server v2.0 (https://services.healthtech.dtu.dk/services/TMHMM-2.0/).

Based on the HOs released in GenBank, a phylogenetic tree was constructed by MEGA v7.0 using the Neighbor-Joining (NJ) algorithm, and 1,000 bootstrap replications were performed. The conserved protein domains of HOs were predicted by using NCBI and the motifs were analyzed by the MEME (https://meme-suite.org/meme/tools/meme). The phylogenetic relationship, motifs and conserved domains of HOs were demonstrated by TB tools (Lu et al., 2020).

### 2.4 Heterologous synthesis of BV

The NhHO1 sequence synthesized by the Sangon Biotech (Shanghai) Co., Ltd. was cloned into the expression vector pETDuet-I, resulting in the plasmid pED-NhHO1. The ApHO1 was cloned from the vector provided by Professor Zang and linked to the vector pETDuet-I, resulting in the plasmid pED-ApHO1. Then the *E. coli* strain BL21 was transformed with pETDuet-I (empty vector), pED-NhHO1 and pED-ApHO1 respectively. The **Supplementary Table S2** listed the detailed information of these recombinant plasmids.

The transformants were cultivated at 37°C with shaking in Luria-Bertani (LB) medium containing 100 µg·mL^–1^ of ampicillin until OD_600_ reached about 0.6. Flasks containing the cultures were supplemented with IPTG at a final concentration of 0.1 mM. After incubation at 18°C for a further 24 h with vigorous shaking, the cells were harvested by centrifugation at 12,000 rpm and 4°C for 10 min.

### 2.5 Heterologous synthesis of PEB

The NhPebA and NhPebB sequence synthesized by the Sangon Biotech (Shanghai) Co., Ltd. was cloned into the expression vector pRSFDuet-I, resulting in the plasmid pRD-NhPebA-NhPebB. The PebS sequence synthesized by the Sangon Biotech (Shanghai) Co., Ltd. was cloned into the expression vector pED-ApHO1, resulting in the plasmid pED-ApHO1-PebS. The *E. coli* strain BL21 was transformed with pED-ApHO1-PebS. Then the *E. coli* strain BL21 contained peD-ApHO1 was transformed with pRD-NhPebA-NhPebB. The **Supplementary Table S2** listed the detailed information of these recombinant plasmids. Then the methods of cell culture and collection are the same as above.

### 2.6 Western blot results of recombinant proteins

The cell pellets were resuspended in a buffer containing 20 mM sodium phosphate, 150 mM NaCl and 5% glycerol at pH 7.5. Cells were lysed by sonication, and cell debris was removed by centrifugation at 12,000 rpm for 5 min. The supernatant was tested for the presence of the target protein by Western blots, after separated by 12% sodium dodecyl sulphate-polyacrylamide gel electrophoresis (SDS-PAGE).

### 2.7 Extraction of BV and PEB

The cell pellets were resuspended in methanol. Cells were lysed by sonication (5s/10s; 15 min), and cell debris was removed by centrifugation at 12,000 rpm for 10 min. The supernatant was sent to the Analysis & Detection Center, Institute of Oceanology, Chinese Academy of Sciences (IOCAS) for LC-MS analysis. The supernatant was load on to Poroshell 120 EC-C18 column (50 mm × 3.0mm, 2.7 µm, Agilent, USA) on a UHPLC machinery (Agilent 1260), with the flow rate 0.3 mL min^-1^ at 30°C. The mass spectrometry analysis was performed employing electrospray ionization in positive ion mode. The eluent was introduced into the high-definition mass spectrometer (Bruker Maxis plus, Germany) analysis. The optimal conditions were as follow: the MS range was m/z 50 - 2000, the desolvation gas temperature was 200°C, the desolvation gas flow was 10 L min^-1^.

## 3 Results

### 3.1 Sequence analysis of NhHO1, ApHO1, NhPebA, NhPebB and PebS genes

The sequence features of NhHO1, ApHO1, NhPebA, NhPebB and PebS are summarized in **Supplementary Table S3**. **Supplementary Table S3** listed the ORF, molecular weight (MW), isoelectric point (pI) of these sequences.

**Supplementary Figure S1** showed multiple sequence alignment results of NhHO1 with ApHO1. From the result, the two sequences have a low similarity. According to the conserved protein domains analysis by NCBI, the HemeO (red line) of NhHO1 was retrieved.

### 3.2 The HOs from Rhodophyta have the unique evolution

**Figure 1:**
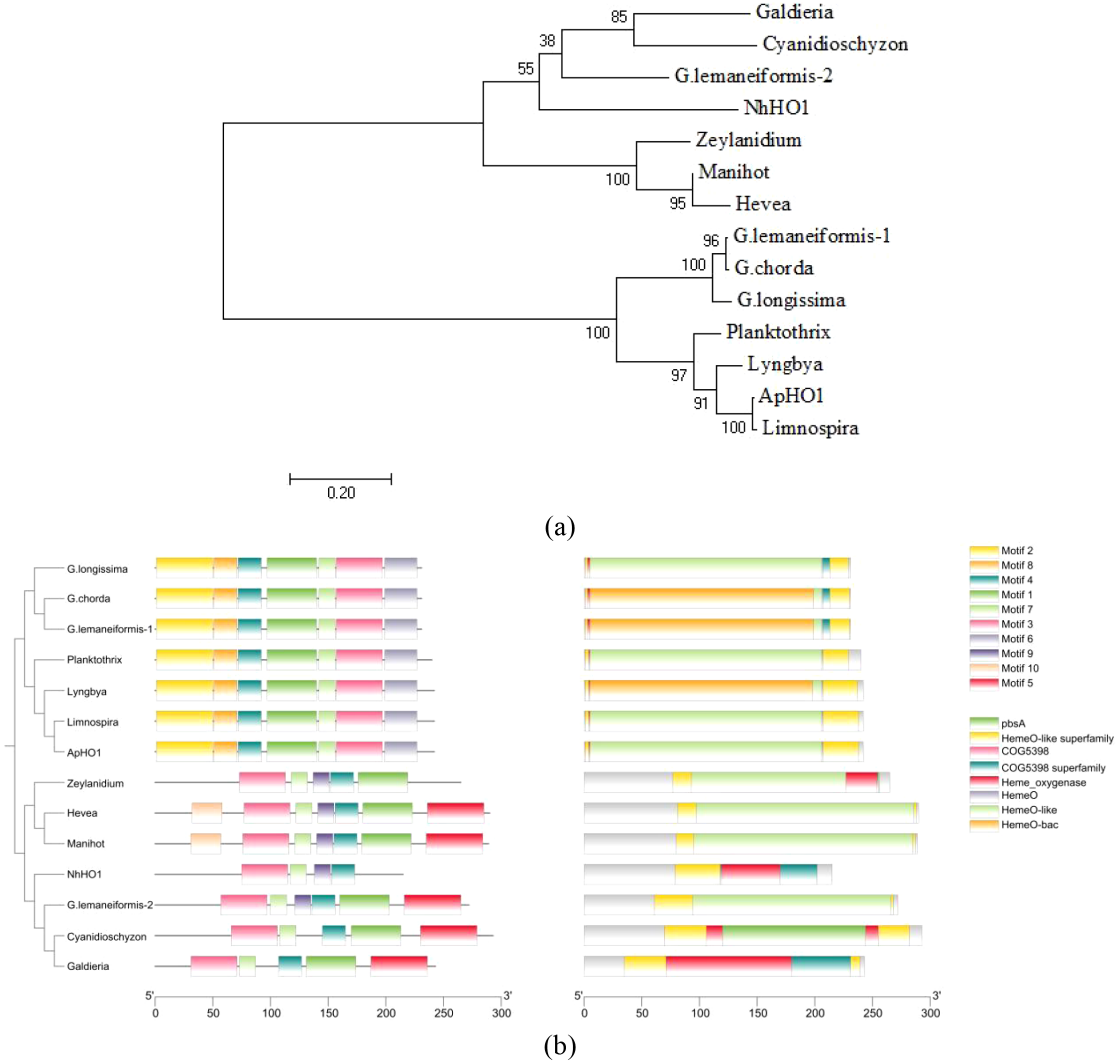
Phylogenetic relationship, motifs and conserved domains of the HO proteins. **(a)** Phylogenetic tree constructed based on HO protein sequences. The tree was constructed using the Neighbor-Joining algorithm with 1,000 bootstrap replicates. The bootstrap value is shown at each node. **(b)** Phylogenetic relationship, motifs (Ten putative motifs were indicated by boxes of different color.) and conserved domains of the HO proteins.

The Neighbor-Joining (NJ) phylogenetic tree was constructed using the HOs amino acid sequence downloaded from NCBI. According to the results of the phylogenetic tree, the HOs from Magnoliopsida, Rhodophyta and Cyanobacteria were clustered into two groups (**Figure 1a**). It is worth mentioning that HOs in Rhodophyta were divided into two clades, one is closer to HOs in Magnoliopsida, while the other is closer to Cyanobacteria. Similarly, in terms of motifs and conserved domains, the HOs from Rhodophyta were also divided into two groups (**Figure 1b**).

### 3.3 Expression of NhHO1, ApHO1, NhpebA, NhpebB and pebS

**Figure 2:**
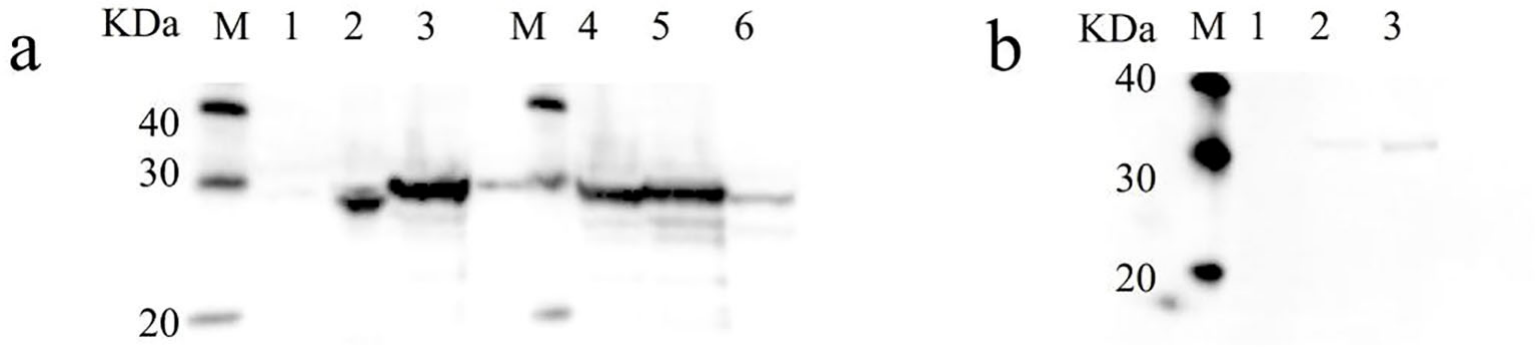
Western blot results of recombinant NhHO1, ApHO1, NhPebA, NhPebB and PebS. **(a)** Western blot results of recombinant NhHO1, ApHO1 and NhPebA. M: protein ladder, Lane 1: crude enzyme extracted from *E. coli* BL21 (petDuet-I), Lane 2: crude enzyme extracted from *E. coli* BL21 (pED-NhHO1), Lane 3 and 4: crude enzyme extracted from *E. coli* BL21 (pED-ApHO1), Lane 5: crude enzyme extracted from *E. coli* BL21 (pED-ApHO1 & pRD-NhPebA-NhPebB), Lane 6: crude enzyme extracted from *E. coli* BL21 (pED-ApHO1-PebS). **(b)** Western blot results of recombinant NhPebB and PebS. M: protein ladder, Lane 1: crude enzyme extracted from *E. coli* BL21 (pED-ApHO1), Lane 2: crude enzyme extracted from *E. coli* BL21 (pED-ApHO1 & pRD-NhPebA-NhPebB), Lane 3: crude enzyme extracted from *E. coli* BL21 (pED-ApHO1-PebS). Original Western blot results are shown in the **Supplementary Figure S2**.

The MW of NhHO1 fusion proteins was about 24 kDa, and the MW of ApHO1 fusion proteins was about 29 kDa. Recombinant His-tagged NhHO1 and ApHO1 were induced to express in *E. coli*, and the western blots analysis confirmed the presence of proteins with the expected sizes (**Figure 2a**). The MW of NhPebA fusion proteins was about 30 kDa, which is close to MW to ApHO1. Consequently, these two proteins exhibit comparable MWs, rendering their corresponding bands indistinguishable in the gel (**Figure 2a**). The both MW of NhPebB and PebS fusion proteins were about 29 kDa. **Figure 2b** confirmed the successful expression of recombinant S-tagged NhPebB and PebS proteins with the expected sizes.

### 3.4 Extraction of BV and PEB

**Figure 3:**
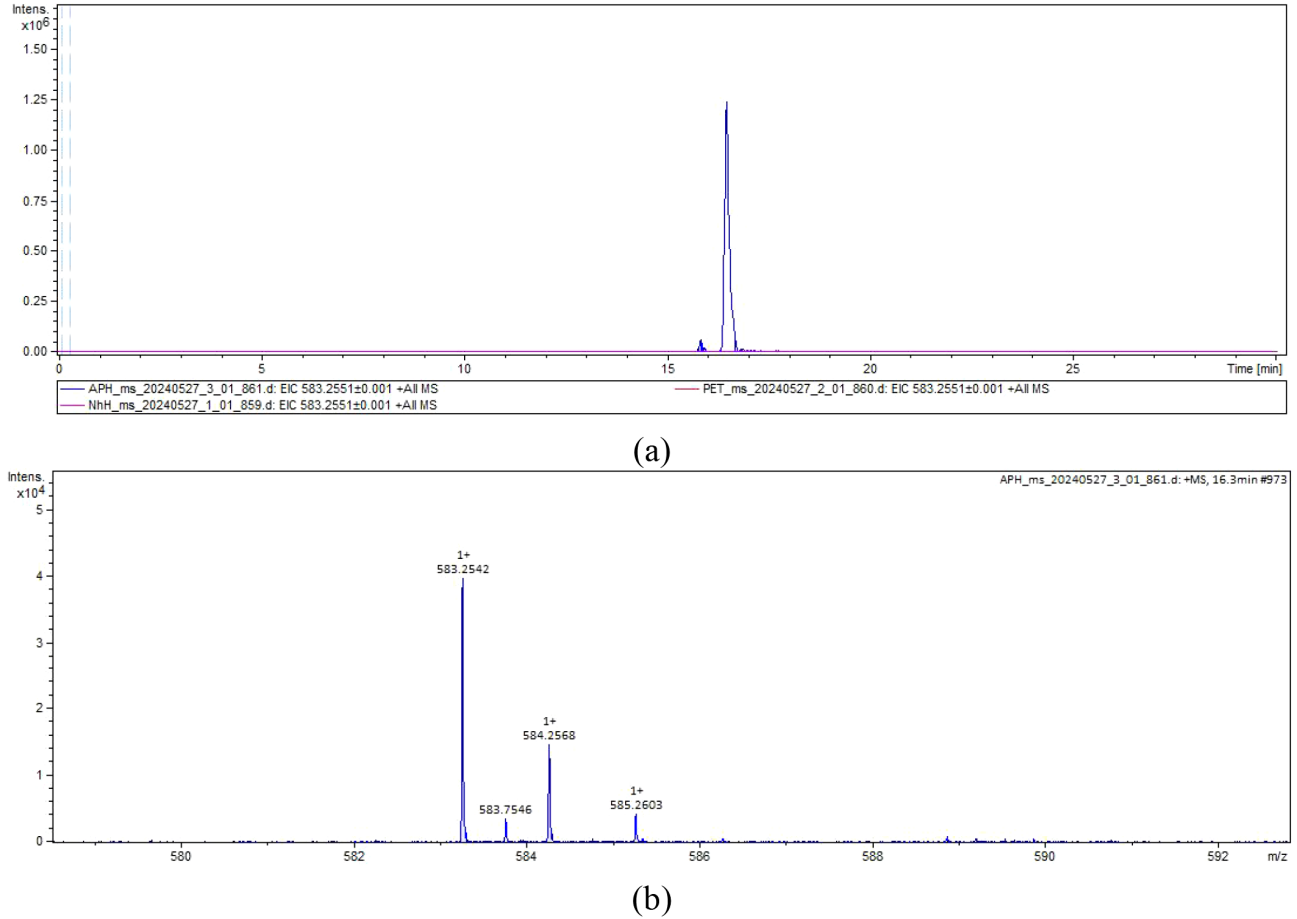
The liquid chromatography-mass spectrometry chromatograms. **(a)** The extracted ion chromatogram. NhH (pED-NhHO1) and APH (pED-ApHO1) were taken as the experimental group (purple and blue curves), and PET (pETDuet-1) was taken as the control group (red curves). **(b)** The mass spectrum. Note the molecular ion species formed: [M + H]^+^.

The molecular weight of the extraction products of *E. coli* BL21 was analyzed by LC-MS. A distinct chromatographic peak appeared in the extraction of *E. coli* BL21 (pED-ApHO1) (**Figure 3a**). Subsequent MS analysis of this peak (**Figure 3b**) confirming the successful synthesis of the BV (C₃₃H₃₄N₄O_6_) (**Figure 3**).

**Figure 4:**
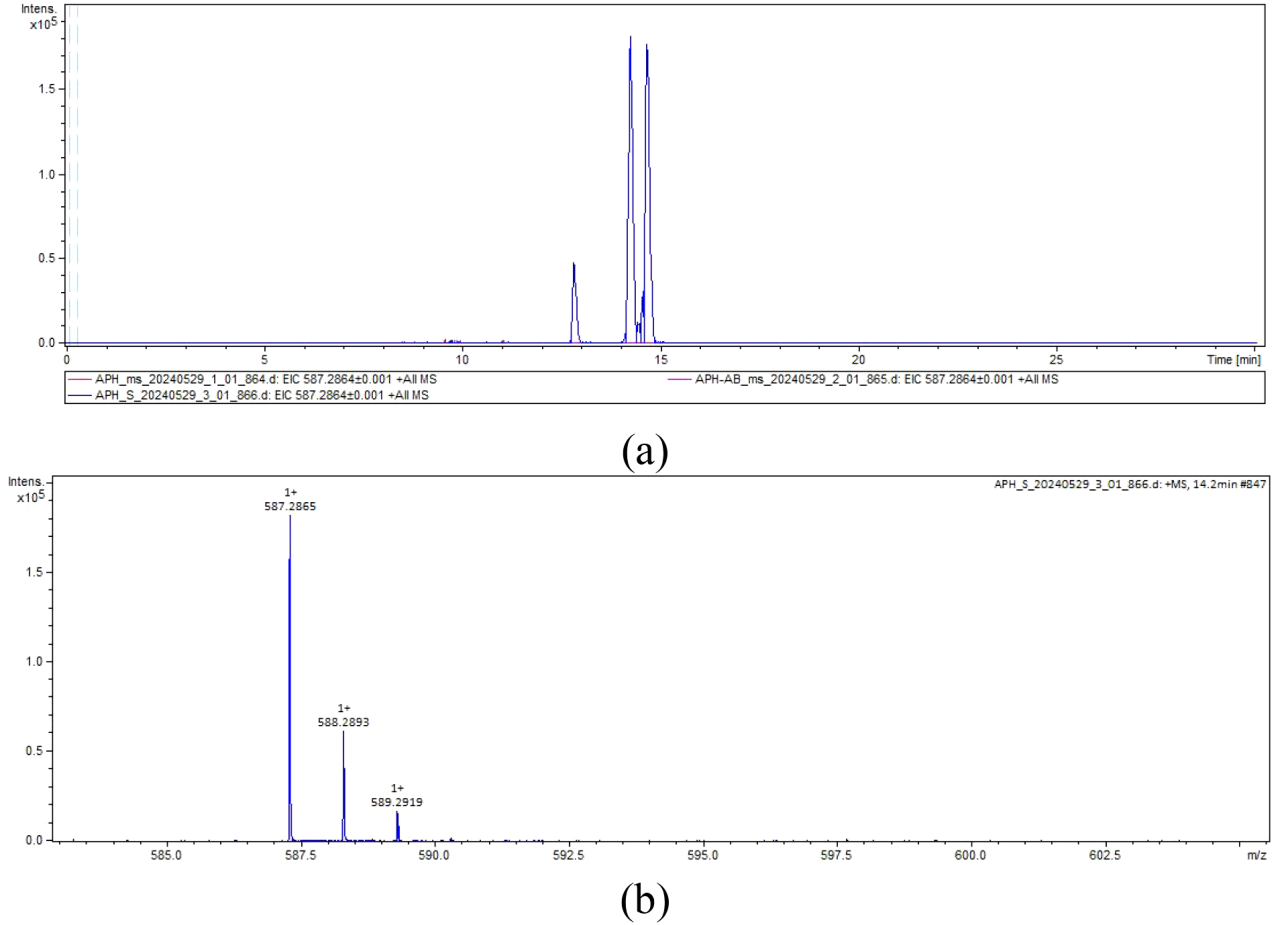
The liquid chromatography-mass spectrometry chromatograms. **(a)** The extracted ion chromatogram. ApH-AB (pED-ApHO1 & pRD-NhPebA-NhPebB, purple curve) and APH-S (pED-ApHO1-PebS, blue curve) were taken as the experimental groups, and ApH (pED-ApHO1) was taken as the control group (red curves). **(b)** The mass spectrum. Note the molecular ion species formed: [M + H]^+^.

Since pED-ApHO1 has been verified to be capable of producing BV, we then used this strain to express NhPebA, NhPebB and PebS. However, NhPebA coupling with NhPebB showed no activity catalyzing the production of PEB (**Figure 4**). In the strain containing recombinant pED-ApHO1-PebS, the characteristic peak appeared in the extraction of *E. coli* BL21 cells (**Figure 4a**). MS analysis of this peak (**Figure 4b**) confirming successful synthesis of PEB (C₃₃H₃₈N₄O₆) (**Figure 4**).

## 4 Discussion

### 4.1 ApHO1 other than NhHO1 induced the biosynthesis of BV in *E. coli*

BV has gained significant interest owing to its diverse applications across medical and material sciences (Seok et al., 2019). At present, environmental unfriendliness and the production of impurities constrained the extraction and purification of BV (Seok et al., 2019). Therefore, the synthesis of BV in *E. coli* would be a simple and efficient way. In this study, the NhHO1 and ApHO1 were introduced into *E. coli* to achieve heterologous synthesis of BV.

**Figure 3** showed that ApHO1 can catalyze the production of BV, whereas NhHO1 exhibits no detectable enzymatic function. Evolutionary relationships and sequence structure of these two HO1s were compared and analyzed. Based on the conserved domain, HOs can be categorized into two main categories, which was in accordance with Jin’s study (2018). The two HOs from *Gracilariopsis lemaneiformis* exhibit distinct conserved domains and are positioned on separate branches of the phylogenetic tree (Jin et al., 2018). Here, the two HO sequences were also selected for analysis along with HOs sequences from other species. According to the phylogenetic tree, HO-2 of *G. lemaneiformis* and NhHO1 were clustered together with land plants. Both of them contained conserved domains similar to those of land plants. ApHO1 was grouped in another branch, which was clustered with HO-1 of *G. lemaneiformis*. Since both HO-1 and HO-2 of *G. lemaneiformis* were active (Jin et al., 2018), the distinct location of NhHO1 and ApHO1 in the phylogenetic tree may not be the reason for the inactivity of NhHO1. The conserved domains predicted in NCBI showed no loss of conserved domains in NhHO1 compared to other HOs. Moreover, NhHO1 has the additional conserved domains compared to HO-2 of *G. lemaneiformis*. However, NhHO1 appears to be missing two motifs compared to other HOs in the same branch, which may contribute to its catalytic inactivity.

Following induction of various bacterial groups, only the bacteria pellets of APH group (pED-ApHO1) exhibited a faint green coloration. LC-MS detection also verified the presence of BV in the induced bacteria. Previous studies have demonstrated the enzymatic activity of HO from various sources, including *Medicago sativa* L (Fu et al., 2011), *Arabidopsis thaliana* (Gisk et al., 2010), *Campylobacter jejuni* (Zhang et al., 2011) and other species. Jin et al. (2012) reported that the recombinant *Brassica rapa* subsp. pekinensis HO1 (BrHO1) expressed in *E. coli* can convert heme into BV. In addition, BrHO1 plays an important role in abiotic stress response (Jin et al., 2012). Muramoto et al. (2002) reported that in the presence of reduced ferredoxin, AtHO1 can catalyze heme to produce BV, which was accompanied by the production of carbon monoxide. They also showed that the basic mechanism of heme cleavage between plants and other organisms is conserved, despite large differences in function, subcellular localization and cofactor requirements of heme oxygenase (Muramoto et al., 2002).

To achieve large-scale biosynthesis of BV, whole-cell transformation or direct synthesis in the chassis cells (e.g. *E. coli*) can be chosed. Yan et al. (2022) successfully engineered recombinant *E. coli* with HO from *Clostridium tetani* and endowed it the ability to transform heme and synthesize BV. Furthermore, the NADPH coenzyme regeneration system and a membrane surface display system were constructed, which shortened the conversion time and improved the BV yield (Yan et al., 2022). So far, there have been some reports on the heterologous synthesis of BV. Chen et al. (2012) constructed recombinant plasmid containing HO from cyanobacteria and transformed it into *E. coli* strain BL21, achieving the large-scale production, recovery and purification of BV. These studies provide a foundation for further optimizing the catalytic performance of ApHO1 to enhance BV synthesis and address the challenge of BV production in *E. coli* strain expressing NhHO1, which currently lacks activity.

### 4.2 ApHO1 and PebS induced the biosynthesis of PEB in *E. coli*

PEB has been widely used in various fields, such as food and cosmetics (non-toxic colorant) (Stiefelmaier et al., 2018). Stiefelmaier et al. (18) described a method for the production and purification of PEB in *E. coli*. In his study, the yield of PEB was increased by adjusting aeration, induction time, medium composition and adding precursors (Stiefelmaier et al., 2018). Beyond *E. coli*, Heck et al. (2024) introduced PebS into *Synechocystis* and proved the formation of PEB. The PcyA gene in *Synechocystis* sp.PCC 6803 was replaced by PebS gene to achieve the synthesis of PEB (Guo et al., 2023). Unlike *PebS*, the introduction of *pebAB* in marine *Synechococcus* strain resulted in phenotypic instability (Alvey et al., 2011). These strains quickly reverted to wild-type appearance, probably due to strong selection pressure that inactivated *pebAB* expression (Alvey et al., 2011). Conversely, Jin et al. (2018) introduced the *HO*, *PebA* and *PebB* from *G. lemaneiformis* into *E. coli* to enable PEB biosynthesis. Building on these insights, our study explored the heterologous expression of *PebS* alongside *NhPebA* and *NhPebB* from *N. haitanensis*, co-introduced with the previously described *ApHO1* into *E. coli*. However, PEB can only be detected in strains that introduce *PebS*. Several factors may account for these observations: first, the exogenous eukaryotic origin of NhPebA and NhPebB may lead to challenges in maintaining functional expression within a prokaryotic system. Secondly, when three genes are co-transformed, the expression of each gene is reduced compared to when only two genes are introduced. Although the heterologous expression of genes from *N. haitanensis* is barely satisfactory and did not exert the expected function, this study successfully produced BV and PEB through the co-introduction of ApHO1 gene and PebS gene into *E. coli*.

Future optimization efforts will focus on refining the induction protocols to maximize BV and PEB yields within the *E. coli* system. Additionally, new genes will be retrieved and identified from *N. haitanensis* for their potential application in BV and PEB biosynthesis. This study facilitates the advancement of heterologous biosynthesis pathways for PE and provides a reference for exploring the application of enzymatic resources derived from economically valuable red algae in the biosynthesis of PE.

## Data Availability

The datasets presented in this study can be found in online repositories. The names of the repository/repositories and accession number(s) can be found in the article/Supplementary Material.
