# Supplementary material for: Systems metabolic engineering of Escherichia coli for the bioproduction of biliverdin and phycoerythrobilin
Source: Front Plant Sci. 2025 Jul 25;16:1640158. doi: 10.3389/fpls.2025.1640158 (PMC12331624; doi:10.3389/fpls.2025.1640158)
Supplement: Supplementary file 1 [file Presentation1.pptx]

## Slide 1
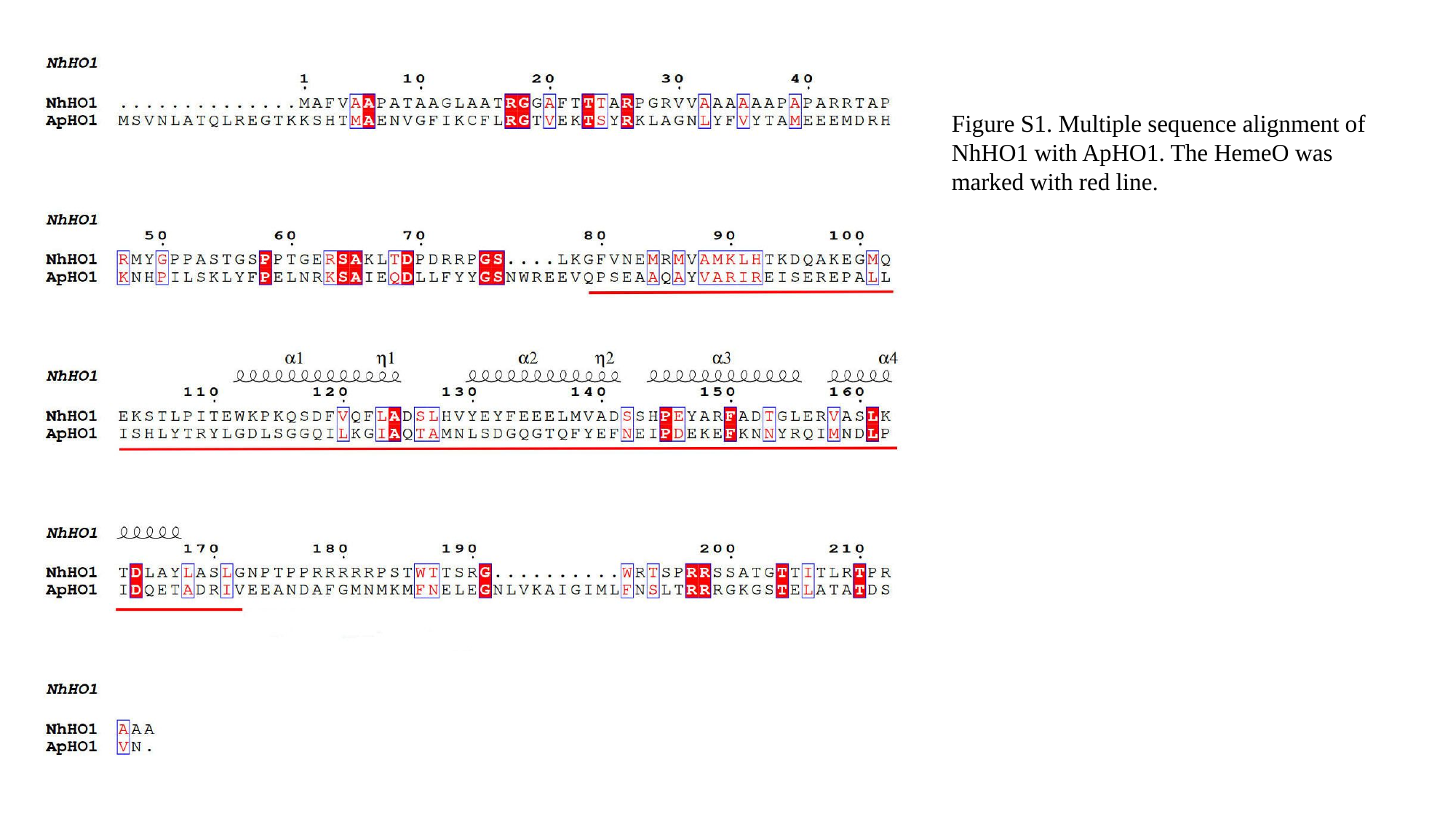

Figure S1. Multiple sequence alignment of NhHO1 with ApHO1. The HemeO was marked with red line.

## Slide 2
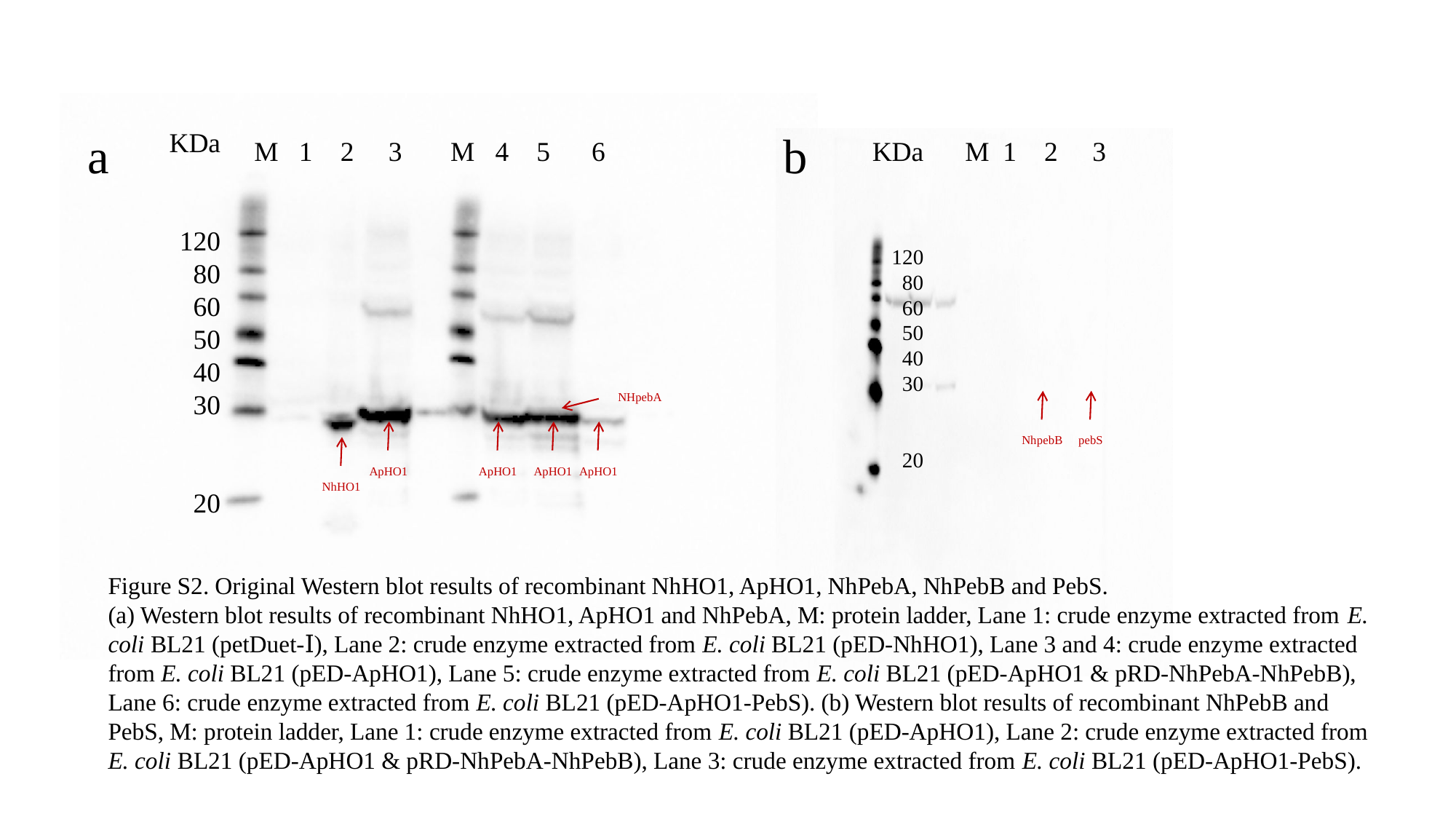

KDa
120
80
60
50
40
30
20
M 1 2 3 M 4 5 6
NHpebA
ApHO1
ApHO1
ApHO1
ApHO1
NhHO1
a
b
KDa
120
80
60
50
40
30
20
M 1 2 3
NhpebB
pebS
Figure S2. Original Western blot results of recombinant NhHO1, ApHO1, NhPebA, NhPebB and PebS.
(a) Western blot results of recombinant NhHO1, ApHO1 and NhPebA, M: protein ladder, Lane 1: crude enzyme extracted from E. coli BL21 (petDuet-Ⅰ), Lane 2: crude enzyme extracted from E. coli BL21 (pED-NhHO1), Lane 3 and 4: crude enzyme extracted from E. coli BL21 (pED-ApHO1), Lane 5: crude enzyme extracted from E. coli BL21 (pED-ApHO1 & pRD-NhPebA-NhPebB), Lane 6: crude enzyme extracted from E. coli BL21 (pED-ApHO1-PebS). (b) Western blot results of recombinant NhPebB and PebS, M: protein ladder, Lane 1: crude enzyme extracted from E. coli BL21 (pED-ApHO1), Lane 2: crude enzyme extracted from E. coli BL21 (pED-ApHO1 & pRD-NhPebA-NhPebB), Lane 3: crude enzyme extracted from E. coli BL21 (pED-ApHO1-PebS).
